# Digital memory assessments and plasma pTau217 enable efficient preclinical Alzheimer’s trials

**DOI:** 10.1016/j.tjpad.2026.100503

**Published:** 2026-02-06

**Authors:** Casey R. Vanderlip, Daniel L. Gillen, Joshua D. Grill, Craig E.L. Stark

**Affiliations:** a Department of Neurobiology and Behavior, 1424 Biological Sciences III Irvine, University of California Irvine, Irvine, CA, 92697 USA; b Department of Statistics, University of California Irvine, Irvine, CA, 92697 USA; c Department of Psychiatry and Human Behavior, University of California Irvine, Irvine, CA, 92697 USA

**Keywords:** Digital memory assessments, pTau217, Alzheimer’s disease, Clinical trials, Prevention, Early detection

## Abstract

**Background:**

Preclinical Alzheimer’s disease (AD) trials enroll cognitively unimpaired, amyloid-positive older adults; however, most remain clinically stable over typical trial durations. Limited near-term decline reduces statistical power and drives large sample sizes and high costs. Scalable enrichment strategies capable of identifying individuals most likely to decline are critically needed.

**Objectives:**

To determine whether a brief digital memory assessment (DMA) and plasma phosphorylated tau 217 (pTau217), individually or combined, identify preclinical AD participants at elevated risk for cognitive and biological progression, and whether such enrichment reduces clinical trial sample-size requirements.

**Design:**

Analysis of data from the Anti-Amyloid Treatment in Asymptomatic Alzheimer’s Disease (A4) Study, a multicenter randomized clinical trial with 240 weeks of follow-up.

**Setting:**

Secondary-prevention trial conducted across sites in the United States, Canada, Australia, and Japan.

**Participants:**

A total of 1,169 cognitively unimpaired adults aged 65–85 years who were amyloid-positive and completed both a baseline DMA and plasma pTau217 measurement.

**Measurements:**

Primary outcome was change in the Preclinical Alzheimer Cognitive Composite (PACC) over 240 weeks. Secondary outcomes included Clinical Dementia Rating–Sum of Boxes, Mini-Mental State Examination, Cognitive Function Instrument, and annualized change in amyloid PET, plasma pTau217, and tau PET.

**Results:**

Participants with both elevated pTau217 and low DMA exhibited the greatest cognitive decline and reached the 240-week PACC decline of the overall cohort 83 weeks earlier. Participants with neither marker showed minimal decline. Dual enrichment reduced sample-size estimates for a clinical trial from 3,252 to 818 participants per arm (75 % reduction). These individuals also demonstrated faster increases in plasma pTau217 and neocortical tau PET.

**Conclusions:**

A brief DMA combined with plasma pTau217 identifies a subset of cognitively unimpaired, amyloid-positive older adults at highest risk for cognitive and biomarker progression. This dual-marker enrichment strategy enables smaller, shorter, and more cost-efficient preclinical AD trials and supports more targeted evaluation of preventive therapies.

## 1 Introduction

Alzheimer’s disease (AD) is characterized by a preclinical phase in which amyloid (Aβ) pathology accumulates for decades before the onset of cognitive impairment [1,2]. This early window, before widespread neurodegeneration, is also the time when disease-modifying therapies may be most effective [3]. These observations have led to a rise in secondary prevention, or preclinical AD, trials [4,5]. The A4 Study, a large preclinical AD trial, demonstrated the feasibility of enrolling Aβ+ cognitively unimpaired (CU) older adults [5,6]. However, A4 also revealed a challenge, within the four- to five-year timeframe of a trial, most individuals with preclinical AD do not exhibit measurable cognitive decline [5,7].

Lack of near-term decline in preclinical AD has profound implications for trial feasibility. From a statistical standpoint, Aβ+ “non-decliners” increase the required sample size needed to demonstrate slowing of cognitive decline. Conversely, those at greatest risk for imminent cognitive decline improve trial feasibility by increasing statistical power.

Plasma pTau217 has emerged as one of the most promising tools for enrichment. It correlates strongly with both Aβ and tau pathology, scales to large populations, and substantially reduces reliance on costly and burdensome PET imaging [8,9]. Plasma pTau217-based screening can be used prior to amyloid confirmation to enrich for individuals highly likely to be Aβ-positive, minimizing negative PET scans in those expected to be ineligible. One trial has used plasma pTau217 as the sole biomarker enrollment criteria [10]. Once Aβ positivity is established, plasma pTau217 levels may also provide complementary information about disease stage and progression risk [11,12]. However, elevated pTau217 alone does not guarantee near-term decline, as many individuals with high levels remain cognitively stable for years [13].

Digital memory assessments (DMAs) provide complementary information [[14], [15], [16]]. These tasks have been designed with a modern understanding of AD progression, recognizing that the hippocampus is one of the first brain regions affected [17,18]. Advances in our knowledge of hippocampal functions have enabled the development of tasks that better target this circuit, making them especially sensitive to early change [19,20]. A central hippocampal computation assessed by many DMAs is pattern separation, the process by which highly similar inputs are transformed into distinct memory representations [[21], [22], [23]]. This computation is known to be particularly vulnerable in aging and AD, reflecting early dysfunction within hippocampal subfields critical for episodic memory [16,24,25]. In line with this, performance on DMAs that tax hippocampal pattern separation can predict both Aβ and tau pathology, identify individuals at elevated risk for future cognitive decline, and integrate effectively with blood-based biomarkers, such as pTau217, to improve prediction of subsequent decline [[26], [27], [28], [29], [30]]. Further, DMAs can be delivered via tablets or smartphones, do not require a neuropsychologist, and can be self-administered in the home, making them particularly attractive for large prevention trials.

Here, we evaluated the role of combining plasma pTau217 and DMAs as enrichment tools for preclinical AD trials. We tested whether these tools, alone or together, can be used to efficiently exclude those unlikely to demonstrate decline and enrich for those who show decline.

## 2 Methods

### 2.1 Participants

All data were obtained from the A4 clinical trial [3,5,6,31]. A4 was a double-blind, placebo-controlled, 240-week Phase 3 trial evaluating solanuzamab, an anti-Aβ monoclonal antibody, in CU older adults with preclinical AD. The study included 1169 Aβ-positive participants, as determined by florbetapir PET scan results, aged 65 to 85, with an MMSE score above 24 and a Global Clinical Dementia Rating (CDR) score of 0. Additionally, participants completed a brief digital memory assessment at screening and underwent a blood test at baseline. Participants were not excluded for missing DMA or pTau217; however, enrichment group assignment was performed using available data for the corresponding measure. In the analytic cohort, 50 participants were missing screening DMA and 133 were missing baseline plasma pTau217 (50 missing both).

### 2.2 Digital memory assessment

The Computerized Cognitive Composite (C3) is a brief digital memory assessment completed by all A4 participants and has been previously described in detail [26,29,30]. Briefly, it includes three hippocampal memory tasks, the Behavioral Pattern Separation Task (BPST), Face-Name Task (FNAME), and One Card Learning Task (OCL).

The BPST, now known as the Mnemonic Similarity Task (MST), is a hippocampal memory task designed to assess pattern separation [32,33]. In A4, a shortened version of the MST was used. Briefly, during the encoding phase, participants viewed 40 images of everyday objects on a white background, making indoor/outdoor judgments via button press (5 s per image, 0.5 s inter-stimulus interval (ISI)). Immediately afterward, they received instructions for a recognition memory test, in which they classified objects as “old” (identical to a previously seen image), “similar” (a slightly altered version of a studied item, such as a different exemplar or rotation), or “new.” During this phase, participants viewed 60 images (5 s per image, 0.5 s ISI), consisting of 20 exact repeats from encoding (targets), 20 completely novel images (foils), and 20 similar but non-identical images (lures). Unlike the MST, the same images were shown at test in both the target and lure forms. The primary measure of interest was the lure discrimination index (LDI), calculated as the proportion of “Similar” responses to lures minus the proportion of “Similar” responses to foils, adjusting for response bias.

For the FNAME, Participants viewed 12 face-name pairs presented sequentially and judged whether each name “fit” the face to maintain attentiveness [34,35]. After a 12- to 15-minute delay filled with other cognitive tasks, memory was assessed through face recognition, first-letter name recall, and face-name matching (FNMT). Each task was scored out of 12, with FNMT accuracy serving as the primary outcome measure.

OCL is a visual memory task modeled off the MST and is a task that taxes hippocampal pattern separation which is critical for episodic memory [16,36]. In this task, participants are shown a series of playing cards and are asked if they have seen the playing card previously during the task. Four cards are randomly selected to repeat eight times throughout the task. The task consists of 80 trials and the performance outcome is accuracy.

The C3 consisted of one primary outcome from each of the three memory tasks and is calculated as the average of these z-scored outcomes, standardized based on screening data.

### 2.3 Neuropsychological assessments

All participants completed in-person, gold-standard neuropsychological testing, including the Preclinical Alzheimer’s Cognitive Composite (PACC) [37,38]. The PACC comprises four components: the total score on the Free and Cued Selective Reminding Test (FCSRT), delayed paragraph recall from the Logical Memory IIa test (Wechsler Memory Scale), the Digit Symbol Substitution Test (Wechsler Adult Intelligence Scale–Revised), and the MMSE total score. To minimize practice effects, alternate versions of these tests were used at each session. To control for baseline performance, each component score was converted to a z-score by subtracting the baseline mean and dividing by the baseline standard deviation. The PACC score was calculated as the sum of these z-scores, with negative values indicating cognitive decline.

In addition to the PACC, we included complementary clinical measures: the Clinical Dementia Rating Sum of Boxes (CDR-SB) to capture global functional impairment, the MMSE as a widely used screening tool, and the Cognitive Function Instrument (CFI), which integrates self- and study-partner reports of subjective decline. These measures served as secondary outcomes and anchors for evaluating enrichment strategies.

### 2.4 Plasma pTau217

Plasma pTau217 concentrations were quantified using an analytically validated electrochemiluminescence (ECL) immunoassay developed by Eli Lilly. Sample preparation was automated using a high-throughput liquid-handling platform (Tecan Fluent workstation), and detection was performed on a Meso Scale Discovery (MSD) Sector S Imager 600 MM. All assays were conducted on baseline plasma samples in a CAP-accredited, CLIA-certified laboratory under standardized procedures. Additional methodological details regarding assay development, validation, and data processing for the A4 Study have been described previously [39].

### 2.5 Statistical analyses

All analyses were conducted in R (version 4.3.3) using the RStudio integrated development environment. Data manipulation and visualization were performed using ggplot2 (version 3.5.2), and mixed-effects modeling relied on nlme (version 3.1–165). Individuals were split into either non-mutually exclusive or mutually exclusive groups based on plasma pTau217 and memory performance. The non–mutually exclusive framework served as the primary approach and reflects real-world screening scenarios, in which enrichment criteria are applied sequentially and participants may meet more than one criterion. Mutually exclusive groupings were evaluated as a complementary analysis to isolate the incremental contribution of each marker and to characterize individuals meeting neither criterion, who may be informative for trials focused on minimal decline or cognitive stability. For the non-mutually exclusive approach, participants could contribute to more than one enrichment group (e.g., an individual could be classified as both Elevated pTau217 and Low DMA). Groups included elevated pTau217, low DMA, and pTau+DMA (meeting criteria for both enrichment strategies). Analyses were conducted in parallel to the All group, which included all A4 participants without enrichment. For both the primary (i.e. PACC) and secondary (i.e. CDR Sum of Boxes, MMSE, and CFI) outcomes, we estimated decline relative to baseline using LOESS regression with 1,000 bootstrap replicates using a span of 0.7 which was empirically selected to balance curve smoothness with goodness of fit across all outcome measures [40]. Using the bootstrapped replicates, we estimated group differences versus the All cohort at Week 240, and “Weeks Earlier,” defined as the week at which an enriched group reached the same mean level of decline observed in the All group at Week 240. Bootstrapped resampling was used to quantify uncertainty across outcomes. This framework allowed us to examine the additive and overlapping value of DMA and pTau217 enrichment strategies, particularly the performance of individuals who satisfied both criteria.

We contrasted the above results with a mutually exclusive enrichment design in which participants were classified into one of four groups: elevated pTau217 only, low DMA only, pTau+DMA, or neither. This scheme allows for clearer comparison of enrichment strategies without overlap between groups. For each outcome measure, we used mixed-model repeated measures (MMRM) with generalized least squares estimation, progressively adding variance and correlation structures (heterogeneous variance by visit, unstructured or autoregressive correlation across visits) to account for within-subject dependencies. Least-squares means were estimated for each group at each visit, and contrasts were computed versus the neither group, with particular attention to Week 240 as the trial endpoint. Pairwise comparisons among groups were also conducted.

To assess whether enrichment strategies captured individuals with greater pathological progression, we also evaluated longitudinal changes in key imaging and fluid biomarkers. For each participant, we calculated annualized change from baseline to Week 240 in Aβ PET (Centiloids), plasma pTau217, medial temporal lobe (MTL) and neocortical tau PET SUVR. Paired observations (baseline and Week 240) were used to derive per-year changes.

To estimate trial feasibility under different enrichment strategies, we considered two complementary trial-design frameworks. First, as a benchmark, we calculated the per-arm sample size required using a simple endpoint-focused design, in which treatment effects were assessed as the difference in change from baseline to Week 240 (Supplemental Equation 1). For each group, mean and standard deviation of the within person change on the PACC at week 240 were used to compute the minimal detectable difference corresponding to 30 % slowing, assuming two-sided α = 0.05, 80 % power, and 30 % attrition. Standard two-sample formulas were applied to derive per-arm sample size requirements. Inflated values accounting for attrition were reported. This approach provides a conservative reference that is commonly used in trial planning.

Second, to reflect a more realistic longitudinal trial design that leverages repeated measurements and within-subject covariance, we additionally implemented a nonparametric bootstrap simulation to estimate statistical power under realistic longitudinal trajectories. For each replicate, we resampled participant trajectories with replacement and assigned them to either the treatment or control arm. The treatment effect specified to be a 30 % slowing of PACC decline. Simulated datasets were analyzed using a hierarchical cascade of models, beginning with a mixed-model repeated measures (MMRM) approach (Supplemental Equation 2, random intercepts, AR(1) correlation, and variance heterogeneity across visits), and using a simplified ANCOVA at week 240 if convergence failed. To approximate real-world follow-up, we applied uniform dropout at ∼6 % per year. Power was estimated as the proportion of replicates with a significant treatment effect (two-sided α = 0.05) across a grid of per-arm sample sizes. This process was repeated for 1,000 replicates per sample size, and Wilson confidence intervals were calculated for power estimates [41].

We also modeled the efficiency of four trial enrichment strategies for a hypothetical 240-week preventative AD trial, using the PACC as the primary outcome and targeting 30 % slowing of decline with 30 % attrition inflation. Per-arm sample sizes were fixed based on prior power analyses. These scenarios were defined as: (Scenario 1) clinical screening followed by Aβ PET, (Scenario 2) plasma pTau217 followed by clinical screening followed by Aβ PET, (Scenario 3) digital memory followed by clinical screening followed by Aβ PET, and (Scenario 4) digital memory followed by plasma pTau217 followed by clinical screening followed by Aβ PET.

We estimated the efficiency of different enrichment strategies using a screening funnel model that worked backwards from the randomized sample size. Because not all recruited participants qualify for randomization, we then applied empirically derived pass rates from the A4 dataset to determine how many individuals would need to be recruited at the outset to yield the target number randomized. Pass rates were estimated for each enrichment “gate”: membership in the global low-memory tertile on the DMA, plasma pTau217 (≥0.178 pg/mL), passing the baseline clinical screen, and Aβ PET positivity (SUVR ≥1.15). Conditional pass rates were used where appropriate (e.g., Aβ positivity given pTau217 or low DMA status).

## 3 Results

### 3.1 Participants with high plasma pTau217 and low DMA exhibit fastest cognitive decline

**Fig. 1:**
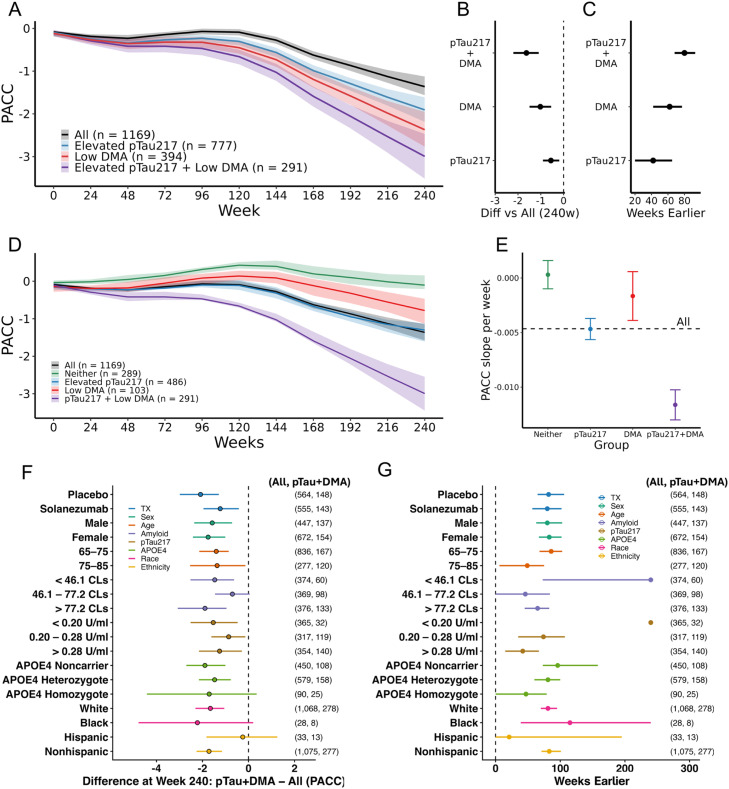
Enrichment with plasma pTau217 and a digital memory assessment identifies individuals most likely to decline. (A) Trajectories of PACC performance over 240 weeks in the overall A4 cohort (black), participants with elevated pTau217 (blue), low DMA performance (red), and both markers (purple). Shaded regions indicate bootstrapped confidence intervals. (B) Differences in PACC decline at Week 240 versus the overall cohort and (C) estimated “weeks earlier,” defined as the earlier timepoint at which enriched groups reached the mean decline of the full A4 cohort at Week 240. (D) Trajectories of PACC performance under mutually exclusive grouping (Neither, pTau217-only, DMA-only, pTau+DMA). (E) Estimated PACC slopes confirmed that the pTau+DMA group declined significantly faster than all others, while the Neither group exhibited little decline. Dashed black line indicates slope for overall cohort. (F-G) Robustness of enrichment effects across treatment, demographic, and biomarker subgroups. Forest plots display (F) bootstrapped differences in PACC decline at Week 240 (pTau+DMA vs. full) and (G) estimated “weeks earlier”. Results are stratified by treatment arm, sex, age, amyloid, plasma pTau217, APOE4 genotype, and race/ethnicity. Across nearly all strata, enrichment with DMA plus elevated pTau217 accelerated decline relative to the broader subgroup, with the pTau+DMA group reaching the A4-average decline up to two years earlier. Ns across all of A4 and the group enriched with pTau+DMA shown to the right of each comparison.

Table 1 describes the participants and Fig. 1 illustrates that, compared to the average trajectory of the full A4 sample, participants with elevated pTau217 declined by an additional 0.56 points over 240 weeks (Fig. 1B, 95 % CI: –0.91 to –0.19), those with low DMA declined by an additional 1.02 points (95 % CI: –1.49 to –0.54), and individuals meeting criteria for both markers declined by an additional 1.63 points (95 % CI: –2.20 to –1.09). These differences translate into efficiency gains when framed as “weeks earlier,” defined as the week at which an enriched group reached the same mean level of decline observed in the All group at Week 240 (Fig. 1C). The elevated pTau217 subgroup reached the same level of decline as the full cohort 63 weeks earlier (95 % CI: 43–73), the DMA alone 71 weeks earlier (95 % CI: 53–80), and the high pTau217 plus low DMA (pTau+DMA) group 83 weeks earlier (95 % CI: 71–98). In other words, the enriched cohorts had greater PACC decline in terms of both magnitude and speed.

**Table 1:** Baseline demographic, clinical, and biomarker characteristics of the A4 cohort and enrichment subgroups. Values are reported as mean (SD) for continuous variables and n (%) for categorical variables. Enrichment groups are non–mutually exclusive.

	All	Elevated pTau217	Low DMA	Elevated pTau217 + Low DMA
n	1,169	777	394	291
Age (years)	71.9 (4.8)	72.4 (4.9)	73.7 (5.3)	74.0 (5.4)
% Female	672 (60%)	464 (60%)	211 (54%)	154 (53%)
Race				
American Indian or Alaskan Native	2 (0.2%)	2 (0.3%)		
Asian	7 (0.6%)	4 (0.5%)	4 (1.0%)	2 (0.7%)
Black or African American	28 (2.5%)	13 (1.7%)	18 (4.6%)	8 (2.7%)
More than one race	8 (0.7%)	6 (0.8%)	4 (1.0%)	3 (1.0%)
Unknown or Not Reported	6 (0.5%)	3 (0.4%)	2 (0.5%)	NA
White	1,068 (95%)	749 (96%)	366 (93%)	278 (96%)
Education (years)	16.61 (2.80)	16.51 (2.80)	16.13 (2.75)	16.09 (2.73)
Ethnicity				
Hispanic or Latino	33 (2.9%)	24 (3.1%)	21 (5.3%)	13 (4.5%)
Not Hispanic or Latino	1,075 (96%)	747 (96%)	371 (94%)	277 (95%)
Unknown or Not reported	11 (1.0%)	6 (0.8%)	2 (0.5%)	1 (0.3%)
MMSE	28.8 (1.3)	28.8 (1.3)	28.4 (1.4)	28.4 (1.5)
APOE Genotype				
E2/E2	2 (0.2%)		1 (0.3%)	
E2/E3	56 (5.0%)	26 (3.3%)	21 (5.3%)	10 (3.4%)
E2/E4	35 (3.1%)	26 (3.3%)	14 (3.6%)	10 (3.4%)
E3/E3	392 (35%)	238 (31%)	147 (37%)	98 (34%)
E3/E4	544 (49%)	413 (53%)	182 (46%)	148 (51%)
E4/E4	90 (8.0%)	74 (9.5%)	29 (7.4%)	25 (8.6%)
Amyloid Centiloids	66 (33)	75 (32)	70 (34)	78 (34)
pTau217 (pg/ml)	0.28 (0.16)	0.32 (0.16)	0.29 (0.15)	0.33 (0.15)
% Solanezumab	555 (50%)	390 (50%)	195 (49%)	143 (49%)

When evaluating mutually exclusive enrichment groups (each participant assigned to only one group) the results were even more striking (Fig. 1D, Supplemental Figure 1). The observed decline on the PACC over four years in the pTau+DMA group exceeded the decline observed in all other groups (Fig. 1E, *p* < 0.001 for all comparisons). Further, only the pTau+DMA group exhibited a faster rate of decline than the entire A4 cohort. These findings suggest that the combination of plasma pTau217 with a DMA identifies a subgroup with the most accelerated decline.

We next examined three additional measures commonly used as AD and preclinical AD trial endpoints, the CDR-SB, MMSE, and CFI, to determine whether patterns of decline extended beyond the PACC (Supplemental Figure 2A–F). In both non-mutually exclusive and mutually exclusive analyses, all three outcomes showed a consistent pattern across groups. Participants in the pTau+DMA group declined most rapidly, showed the greatest difference from the full cohort at 240 weeks, and reached the average four-year decline of the overall A4 cohort roughly 1.5–2 years earlier. Participants with neither elevated pTau217 nor low DMA showed the least change over time.

The observed impact of enrichment for pTau+DMA was consistent across demographic and biomarker subgroups (Fig. 1F-G). Participants in the pTau+DMA group declined more rapidly than their respective parent groups across nearly all strata, including treatment arm, sex, age, APOE genotype, Aβ and pTau217 pathology tertiles, and ethnicity and race. At Week 240, enriched individuals consistently showed greater worsening and reached the average four-year decline of their broader subgroup approximately 1.5–2 years earlier. It is worth noting that these metrics may be misleading for those in the very low Aβ and pTau217 groups, given that these individuals did not exhibit substantial decline over the trial period.

### 3.2 Comparative efficiency of enrichment strategies

**Fig. 2:**
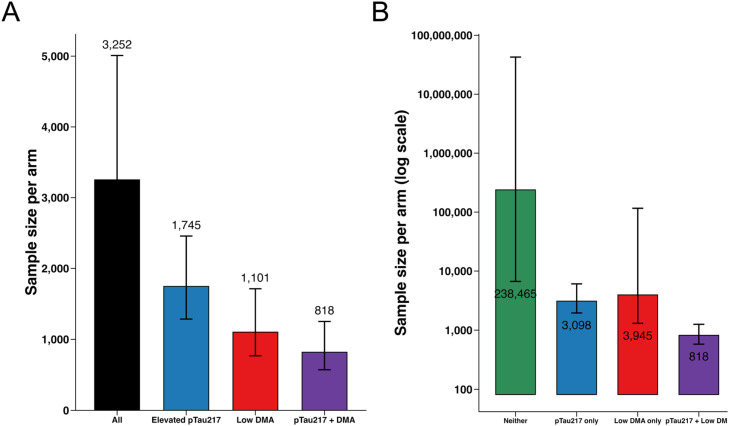
Sample size requirements with different enrichment approaches (A) Sample size calculations for 80 % power to detect a 30 % slowing on the PACC at Week 240 showed that enrichment reduced required per-arm sample sizes compared to the full A4 cohort. (B) Under mutually exclusive classification, the efficiency benefit was driven almost entirely by the pTau+DMA group, whereas pTau217-only and DMA-only required similar or larger samples than the All group. The Neither group would require an unrealistically large sample size.

An important question is whether enriching on plasma pTau217 and/or DMA performance would lower the number of participants needed to detect a 30 % slowing of PACC decline at 80 % power. Sample size estimates were derived from a two-sample difference-in-means framework using observed variability in PACC at Week 240, assuming a 30 % relative treatment effect and 30 % attrition. Required sample sizes per arm were substantially reduced when enrichment was applied. In the non–mutually-exclusive approach, the sample size fell from 3,252 (CI: 2346 -5009) per arm in the full A4 cohort to 1,745 (CI: 1285 -2457) when selecting for elevated pTau217, 1,101 (CI: 769 -1712) for low DMA, and 818 (CI: 572–1252) per arm for those with both risk markers (Fig. 2A). In contrast, the mutually-exclusive strategy showed that this efficiency gain was almost entirely driven by the pTau+DMA group: while the pTau+DMA group required only 818 (Fig. 2B, CI: 579 -1256) participants per arm, the elevated pTau217–only (3,098, CI: 1955–6050) and low DMA–only (3,945, CI: 1304–116053) groups required as many or more participants than the unenriched cohort (3,252, CI: 2346 -5009 per arm). Having neither risk factor was associated with 238,465 (CI: 6655–43282741) participants needed per arm (Fig. 2B).

As a secondary approach that more reliably controls for interindividual variability, we conducted a nonparametric bootstrap simulation that resampled participant trajectories under realistic trial conditions (Supplemental Figure 3A). Results were highly consistent with the standard analytic approach, showing substantial reductions in required sample size with enrichment. Using the full A4 cohort required an estimated 2,718 participants per arm (CI: 2,597–2,847). Enrichment with elevated pTau217 reduced this requirement to 1,378 per arm (CI: 1,295–1,501), while DMA alone enrichment required 945 per arm (CI: 907–1,008). The greatest efficiency was again achieved by combining both markers, with only 741 participants per arm needed (CI: 712–790).

When we repeated the nonparametric bootstrap simulation under the mutually exclusive grouping strategy (Supplemental Figure 3B), the results again confirmed the efficiency of dual enrichment. The unenriched group did not reach 10 % power within the range (4,000 per arm) used. In contrast, the pTau+DMA group reduced the requirement to only 742 per arm (CI: 721–787). By comparison, enrichment on elevated pTau217 without low DMA required 2,523 per arm (CI: 2,421–2,639), and enrichment on low DMA (without high pTau217) required 3,174 per arm (CI: 2,994–3,281).

**Fig. 3:**
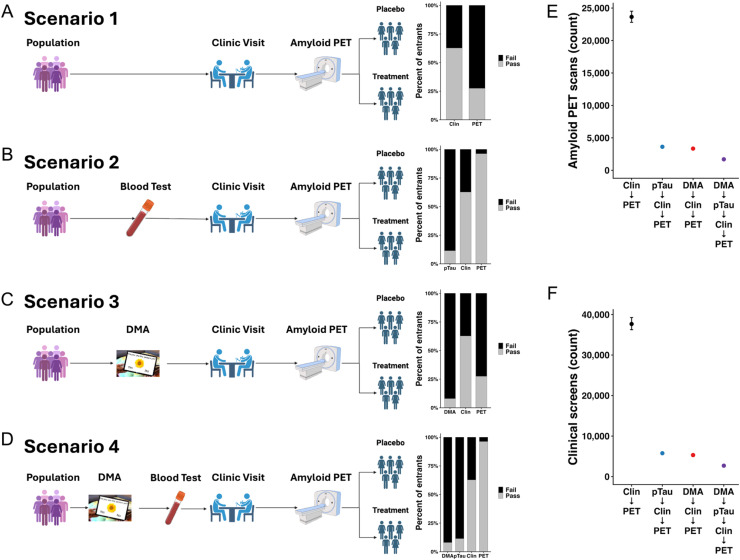
Comparative efficiency of alternative enrichment funnels. (A–D) Schematic screening funnels for four candidate trial designs: (A) traditional clinical visit followed by amyloid PET (Scenario 1), (B) plasma pTau217 → clinical visit → amyloid PET (Scenario 2), (C) digital memory assessment (DMA) → clinical visit → amyloid PET (Scenario 3), and (D) combined DMA → plasma pTau217 → clinical visit → amyloid PET (Scenario 4). Right panels show empirically derived pass/fail rates at each stage based on A4 data, highlighting when attrition occurs along the funnel. E–F) Resource demands across scenarios. The clinical+PET pathway required ∼38,000 clinical visits, ∼25,000 amyloid PET scans, far exceeding enriched designs. The DMA+pTau217 funnel yielded the lowest burden, requiring ∼5,000 clinical visits and ∼2,500 amyloid PET scans.

We next evaluated the efficiency of alternative screening funnels by modeling participant flow through empirically derived pass rates at each screening stage (digital memory, plasma pTau217, clinical screen, Aβ PET). The traditional clinical visit followed by Aβ PET pathway (Fig. 3A) was the least efficient, as most participants passed the initial clinical visit but only about one-third were PET eligible, leading to most exclusions occurring late in the funnel after the most expensive procedures. Adding plasma pTau217 prior to clinical screening (Fig. 3B, scenario 2) improved efficiency by excluding a large subset earlier, thereby reducing the number of participants proceeding to PET. Using DMA as the initial gate (Fig. 3C, scenario 3) shifted the majority of exclusions to the earliest and least expensive stage, reducing the number of individuals requiring clinical and PET assessments. Finally, the combined enrichment strategy (Fig. 3D, scenario 4: DMA to plasma pTau217 to clinical visit to Aβ PET) yielded the most efficient funnel. Exclusions occurred predominantly at the inexpensive DMA and plasma pTau217 stages. Among those reaching Aβ imaging, nearly all were confirmed positive, resulting in minimal late-stage attrition.

We also compared resource requirements across the four screening strategies. The traditional approach of screening directly with clinical assessments followed by Aβ PET was the most resource intensive (Fig. 3E-F). This design required 23,651 Aβ PET scans and 37,684 clinical visits. In contrast, incorporating enrichment gates substantially reduced downstream testing. Strategies beginning with plasma pTau217 or DMAs reduced Aβ PET scans to 3,300–3,700 and clinical visits to ∼5,300–5,800. The combined strategy of DMA plus plasma pTau217 yielded the lowest overall burden, requiring 1,695 Aβ PETs and 2,700 clinical visits. This highlights that simple enrichment gates at screening can reduce both clinical visits and imaging by more than 75 %. Importantly, scenario 4 greatly reduced the number of pTau217 blood tests compared to scenario 2 and did not require more DMAs compared to scenario 3 (Supplemental Figure 4).

### 3.3 Changes in AD biomarkers across enrichment groups

We next asked whether groups differed in biomarker accumulation, focusing first on Aβ PET and plasma pTau217. For Aβ PET, all enrichment groups trended toward faster accumulation compared to the overall sample. The pTau217-enriched group showed a significantly greater annualized rate of Aβ increase (Supplemental Figure 5A-B, 0.0043, CI: 0.0016–0.0071), while both the DMA alone group (0.0020, CI: −0.0016–0.0056) and the pTau+DMA group (0.0030, CI: −0.0013–0.0075) showed numerically higher rates, though confidence intervals included zero. For plasma pTau217, enrichment groups likewise exhibited higher accumulation relative to the full cohort. The pTau+DMA group showed the largest annualized increase (Supplemental Figure 5C-D, 0.0056, CI: −0.0005–0.0126), followed by the DMA group (0.0038, CI: −0.0020–0.0099). The pTau217 group had a smaller unreliable effect (0.0017, CI: −0.0024–0.0055).

We next examined tau PET in the MTL and neocortex in the subset of participants who underwent tau imaging. For MTL tau, the pTau217–enriched group exhibited a significantly greater annualized rate of increase relative to the full cohort (Supplemental Figure 5E-F, 0.0038, CI: 0.0002–0.0077). The pTau+DMA group showed a numerically higher rate (0.0033, CI: −0.0026–0.0093), while the DMA-only group showed no evidence of faster accumulation (−0.0016, CI: −0.0065–0.0036). Neocortical tau showed stronger enrichment effects. Both the pTau217 group (Supplemental Figure 5G-H, 0.0085, CI: 0.0008–0.0170) and the pTau+DMA group (0.0179, CI: 0.0051–0.0331) accumulated neocortical tau significantly faster than the overall A4 cohort. The DMA-only group also trended toward higher rates of neocortical tau accumulation (0.0089, CI: −0.0013–0.0209). Together, these results suggest that enrichment captures individuals at elevated risk for progressive pathology (particularly pTau217 and neocortical tau).

## 4 Discussion

In this study, combining a DMA with plasma pTau217 identified a subgroup of CU, Aβ+ older adults who declined faster, reached the trial-average endpoint years earlier, and required dramatically fewer participants to power a clinical trial. As importantly, Aβ+ individuals who did not meet either criterion were unlikely to decline, underscoring the value of this approach not only for pinpointing those at highest risk, but also for excluding those who would add noise and obscure treatment effects in clinical trials. Together, these findings may inform practical strategies to make preclinical AD trials smaller, shorten the maximal follow-up needed, and increase cost-effectiveness, while also offering a framework for targeting emerging treatments to those most likely to benefit in real-world clinical settings.

Prior work has shown that elevations in plasma pTau217 and poorer performance on DMAs each predict future decline, but with heterogeneity [9,14,26]. Plasma pTau217 offers the advantage of directly reflecting AD pathology, while digital tasks capture subtle cognitive deficits that may foreshadow decline, albeit with less specificity [12,27,39,42]. Our results suggest that the true power lies in combining these complementary approaches. When implemented together, plasma pTau217 and digital memory identified a subgroup of CU, Aβ+ older adults who declined most rapidly over 4.6 years. This pattern was notably consistent across outcome measures and demographic groups and persisted even when accounting for variability in biomarker levels, underscoring the robustness of the combined signal. This group reached the mean decline of the full A4 cohort 83 weeks earlier, a shift that could substantially accelerate and reduce the cost of these trials.

Enrichment with a DMA and plasma pTau217 also translated directly into sizable reductions in the number of participants and cost for adequately powered trials. By restricting to the subgroup with both markers, the sample size needed to detect a reliable treatment effect was reduced by more than 75 %. The gained efficiency was demonstrated using both conventional sample size formulas and nonparametric bootstrapping of actual longitudinal data. Further, in the standard clinical-plus-PET approach used in A4, nearly three-quarters of screened participants failed at the Aβ PET stage, after undergoing the most expensive and burdensome procedure. Our modeling shows that introducing a low-burden DMA as the first screen, followed by a plasma pTau217 test, shifts exclusion to earlier, less expensive stages. With this approach, fewer than 5 % of individuals failed to exhibit elevated Aβ PET.

The results may also instruct efforts to streamline trial recruitment. Trial-ready cohorts are increasingly incorporating digital cognitive assessments, establishing the infrastructure needed to deploy DMAs remotely at scale [[43], [44], [45]]. At the same time, rapid advances in remote collection of plasma biomarkers (e.g. finger-prick sampling) may enable integration of both enrichment tools entirely at home [46]. Such a paradigm would allow investigators to remotely identify participants likely to be trial-eligible before they ever undergo costly or invasive procedures.

Biological endpoints are also gaining traction in AD research, with trials now incorporating Aβ or tau accumulation as secondary or even primary outcomes [4,47,48]. Our findings demonstrate that enrichment with DMAs and plasma pTau217 also identifies those with faster rates of pathobiological progression, particularly in plasma pTau217 and neocortical tau.

As anti-Aβ and anti-tau therapies gain approval for preclinical disease, the central clinical question will move from whether these drugs work to who should receive them and when. Treating every Aβ+ individual may not feasible, at least for treatments requiring repeated infusions and safety monitoring through imaging. As anti-Aβ therapies move earlier in the disease course, a critical policy and ethical debate will center on when treatment confers clear benefit, with payers likely prioritizing those at greatest short-term risk for decline. Our findings point to integrating a DMA with plasma pTau217 to assist clinicians in identifying individuals who are most in need of treatment.

Several limitations should be acknowledged. A4 was a highly educated and primarily non-Hispanic White cohort. Although our subgroup analyses showed consistent enrichment effects across sex, race, APOE genotype, and biomarker-defined strata, replication in more diverse and representative populations is essential. In particular, limited data are currently available on the cross-sectional and longitudinal performance characteristics of plasma pTau217 assays in underrepresented preclinical populations, which may influence enrichment performance across cohorts. This analysis focused on a single digital memory assessment; other digital tasks may capture complementary or even more sensitive aspects of early cognitive change. Moreover, in preclinical disease, treatment benefit may manifest not only as slowing near-term cognitive decline but also as prolongation of cognitive stability, and the factors driving imminent decline may extend beyond amyloid and tau pathology alone. Strategies that rely only on amyloid or tau biomarkers alone may not fully optimize efforts to identify those at greatest risk for imminent cognitive decline. Importantly, while enrichment with pTau and DMA could make prevention trials smaller, faster, and more cost-effective, it would also result in a more selective population being studied and ultimately treated, potentially limiting the generalizability of trial findings and impacting regulatory considerations for treatments shown to be efficacious. Crucially, whether this approach increases false-negative rate (missed decliners) in clinical practice must also be investigated. Careful consideration of this trade-off, between precision in targeting those at highest risk and inclusivity of the broader at-risk population, will be essential.

In summary, combining a DMA with plasma pTau217 may identify Aβ+ CU older adults who are at greatest risk of near-term cognitive and functional decline. This enrichment strategy not only has the potential to reduce trial sample sizes by more than 75 % but also dramatically lowers costs and participant burden by shifting screen failures to inexpensive, scalable tests. Beyond clinical trials, this approach may help inform future clinical decision-making frameworks in the event that disease-modifying therapies are shown to be effective in preclinical disease.

## Sources of funding

This research was funded, in part by R01 AG066683 (CS), P30 AG066519 (CS) and T32AG00096 (CS, CV).

## Consent statement

All human subjects provided informed consent.

## Declaration of generative AI and AI-assisted technologies in the writing process

During the preparation of this work the authors used ChatGPT to find errors in scripts in R. After using this tool/service, the authors reviewed and edited the content as needed and take full responsibility for the content of the publication.

## Data statement

Data from the Anti-Amyloid Treatment in Asymptomatic Alzheimer’s Disease (A4) Study are available to qualified investigators through the A4 Study data sharing platform (https://www.a4studydata.org), subject to data use agreements and study governance procedures. Detailed descriptions of data preparation, harmonization, and sharing procedures are provided in the associated data package and in Jimenez-Maggiora et al. (2024).

## CRediT authorship contribution statement

**Casey R. Vanderlip:** Writing – original draft, Formal analysis, Data curation, Conceptualization. **Daniel L. Gillen:** Writing – review & editing, Methodology. **Joshua D. Grill:** Writing – review & editing, Methodology. **Craig E.L. Stark:** Writing – review & editing, Supervision, Methodology, Funding acquisition.

## Declaration of interest

The authors declare the following financial interests/personal relationships which may be considered as potential competing interests:

Casey Vanderlip reports financial support was provided by National Institute on Aging. Craig Stark reports financial support was provided by National Institute on Aging. Dr. Grill has received funding from the NIA, Alzheimer’s Association, BrightFocus Foundation, Eli Lilly, Biogen, Genentech, and Eisai. He has provided paid consultation to SiteRx, Cogniciti, and FlintRehab. If there are other authors, they declare that they have no known competing financial interests or personal relationships that could have appeared to influence the work reported in this paper.

## Appendix Supplementary materials

Image, application 1
