# Supplementary material for: Digital memory assessments and plasma pTau217 enable efficient preclinical Alzheimer’s trials
Source: J Prev Alzheimers Dis. 2026 Feb 6;13(4):100503. doi: 10.1016/j.tjpad.2026.100503 (PMC12907085; doi:10.1016/j.tjpad.2026.100503)
Supplement: Supplementary file 1 [file mmc1.docx]

**Sample size formulas:**

Eq1:
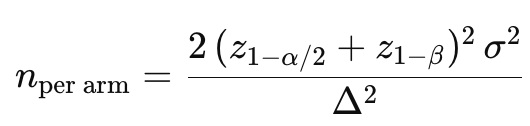


Eq2:
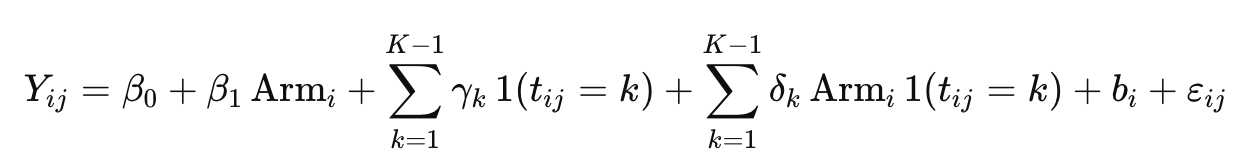


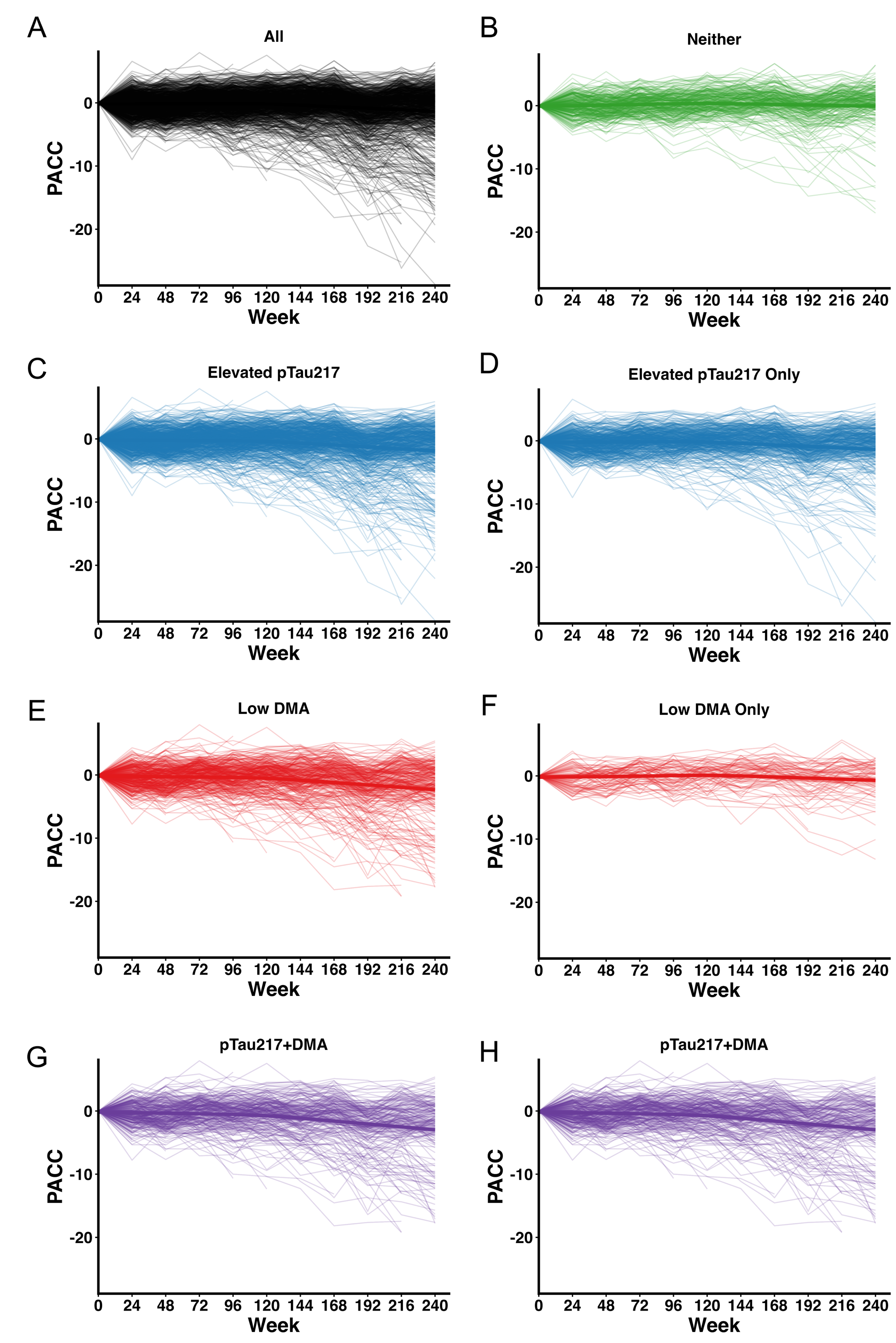
 Supplemental Figure 1. Individual PACC trajectories by non-mutual exclusive and mutually exclusive groups, A) All, B) Neither, C) Elevated pTau217, D) Elevated pTau217 only, E) Low DMA, E) Low DMA only, and G-H) Elevated pTau217 and Low DMA.


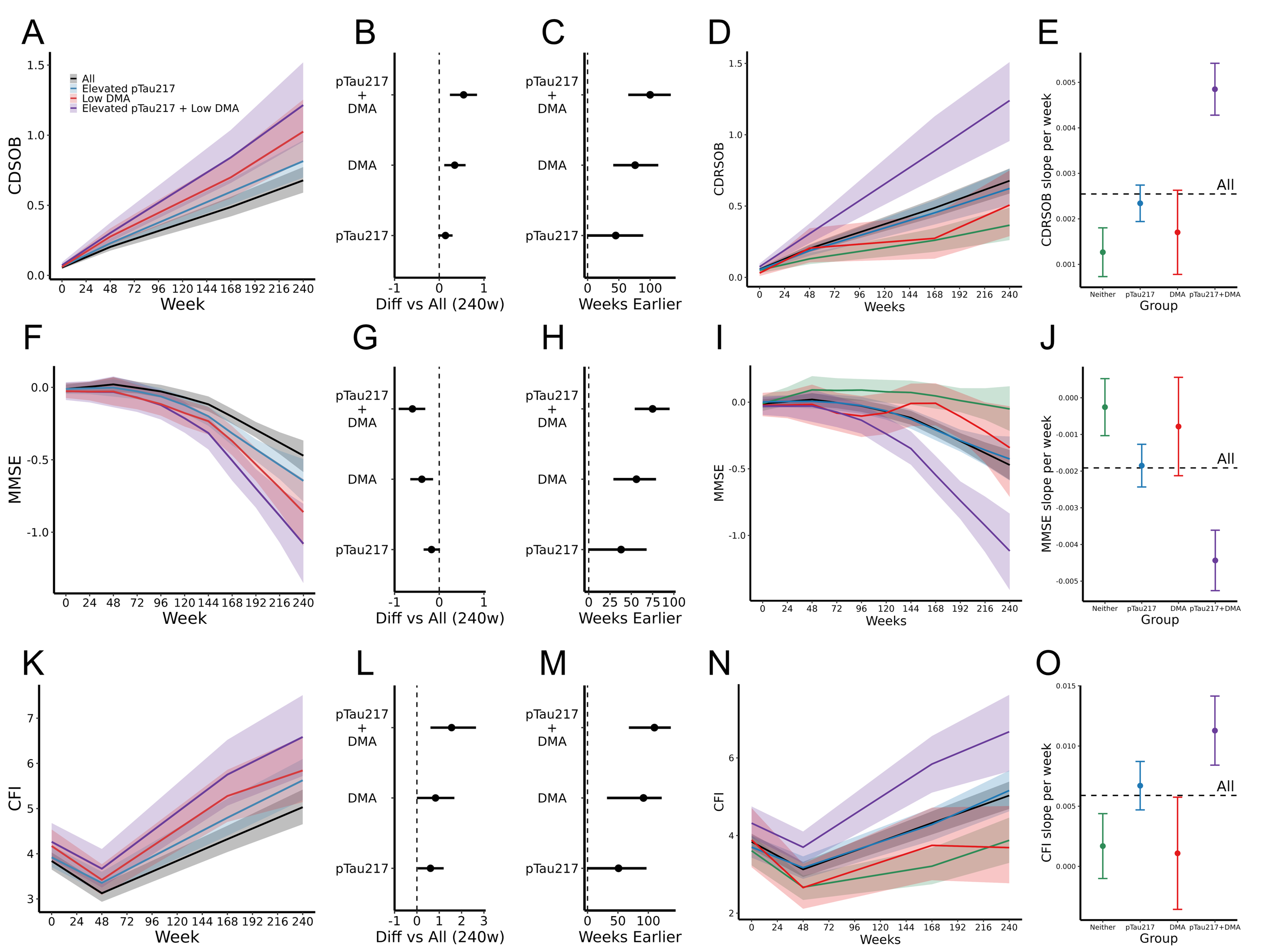
 Supplemental Figure 2. Performance on secondary outcomes after enriching with plasma pTau217 and digital memory assessments (DMA). Longitudinal trajectories of A) CDR-SOB, B) MMSE, and C) CFI over 240 weeks for the full cohort (“All”) and for three non–mutually exclusive enrichment groups: individuals enriched on elevated plasma pTau217, individuals enriched on low DMA performance, and individuals meeting both enrichment criteria. These curves illustrate that enriched groups, particularly those with both elevated pTau217 and low DMA, demonstrate faster worsening compared with the entire sample. Panels B, G, and L display the difference from the All group at Week 240, quantifying how much additional decline each enrichment group shows by the end of follow-up. Panels C, H, and M show how many weeks earlier each enrichment group reaches the clinical endpoint observed in the All group at Week 240, demonstrating acceleration in detectable decline when enriching on pTau217 and/or DMA. Panels D, I, and N present mutually exclusive groups directly compared on their modeled trajectories from a common baseline, reinforcing that combined pTau217 + DMA enrichment yields the steepest and most readily detectable decline. Panels E, J, and O summarize the estimated slopes of change per week for each mutually exclusive group, with the dashed line indicating the slope for the full cohort. Together, these results show that enrichment based on elevated pTau217 and impaired DMA, individually and especially jointly, substantially increases the rate of detectable progression, supporting their use as trial enrichment strategies.


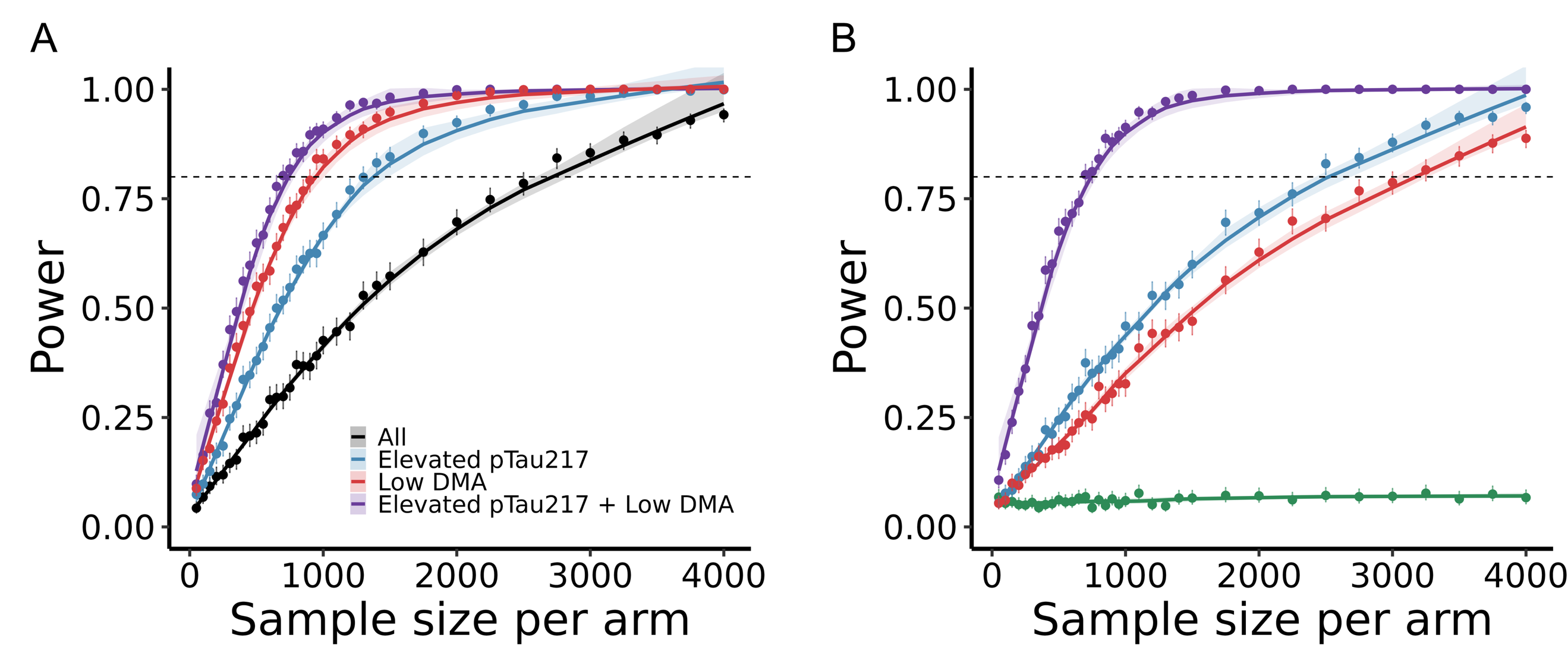
Supplemental Figure 3. Power curves from bootstrap-based clinical trial simulations using pTau217 and DMA enrichment. Panels A and B show statistical power as a function of sample size by different enrichment approaches. Power was estimated using a nonparametric bootstrap simulation that resampled participant trajectories under realistic trial conditions and evaluated treatment effects using an MMRM framework. A. In non-exclusive groups, enrichment on pTau217 and/or DMA markedly increases power across all sample sizes, with the combined enrichment group reaching 80% power at the smallest sample sizes. B) Similar results were found in mutually exclusive groups, demonstrating that recruitment of individuals meeting both enrichment criteria greatly reduces the needed sample size.


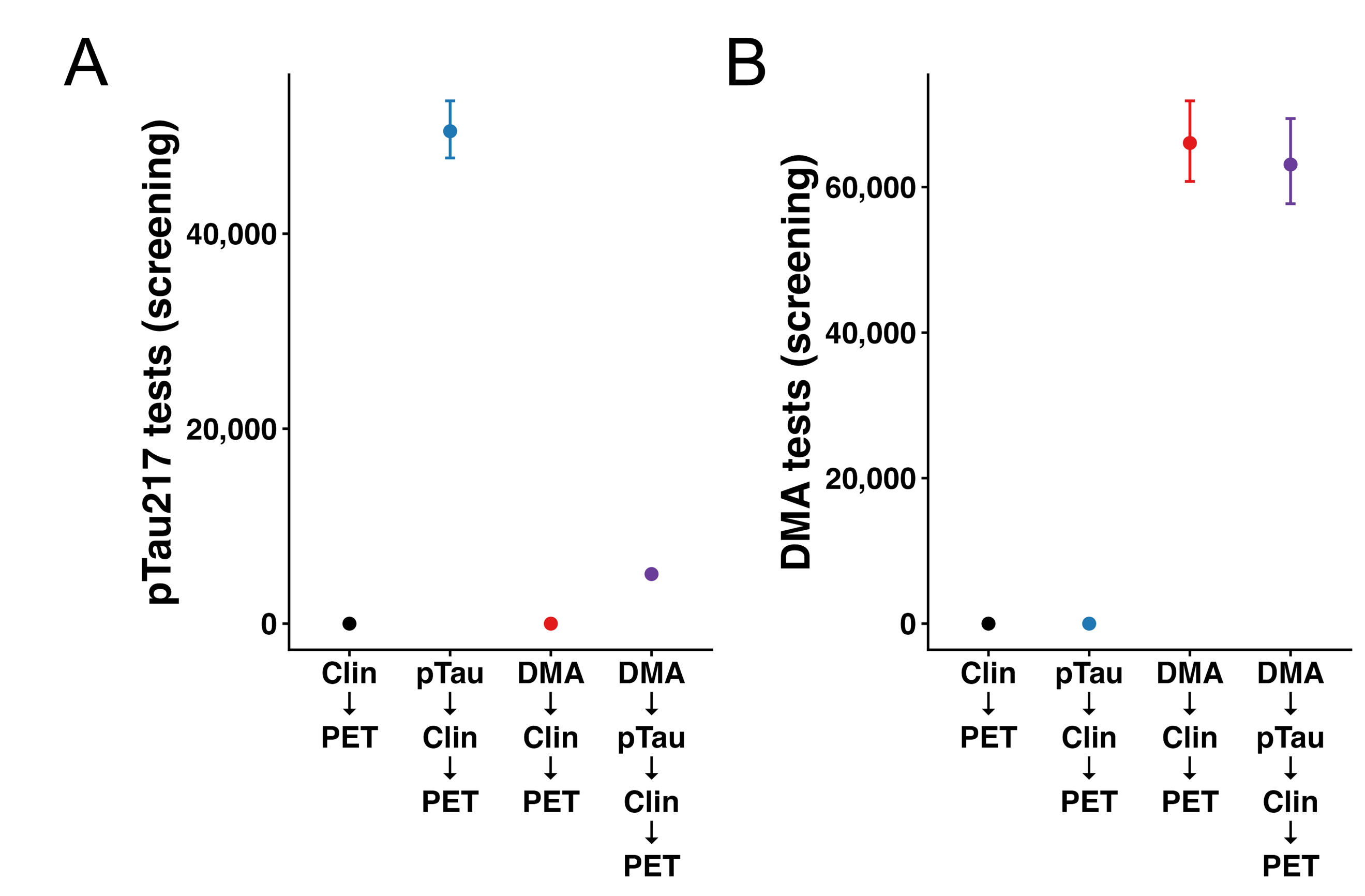
Supplemental Figure 4. Number of pTau217 tests and B) DMAs required across scenarios.

Supplemental Figure 5. Annualized biomarker accumulation across enrichment groups. Violin plots and forest plots show the distribution of annualized change in amyloid PET (A,B) plasma pTau217 (C,D), MTL tau PET (E,F), and neocortical tau PET (G,H) for the full A4 cohort (black), elevated pTau217 (blue), DMA (red), and combined enrichment (purple). White dots indicate group means. Forest plots display bootstrapped mean differences (Δ per year) versus the full cohort, with 95% confidence intervals.
